# Stress, anxiety, and illness perception in patients experiencing delay in operative care due to the COVID-19 pandemic

**DOI:** 10.1016/j.gore.2023.101245

**Published:** 2023-07-13

**Authors:** Michelle A. Soloff, Trey Keel, Aaron Nizam, Gary L. Goldberg, Antoinette Sakaris, Michael A. Diefenbach, Danielle K. DePeralta, Marina Frimer

**Affiliations:** a Division of Gynecologic Oncology, Department of Obstetrics and Gynecology, Northwell Health, Zucker School of Medicine at Hofstra/Northwell, 270-05 76^th^ Avenue, Queens, NY 11040, United States; b Zucker School of Medicine at Hofstra/Northwell, 500 Hofstra Blvd, Hempstead, NY 11549, United States; c Karches Center for Oncology Research, Feinstein Institutes for Medical Research, 350 Community Drive, Manhasset, NY 11030, United States; d Department of Medicine, Northwell Health, Zucker School of Medicine at Hofstra/Northwell, 500 Hofstra Blvd, Hempstead, NY 11549, United States; e Division of Surgical Oncology, Department of General Surgery, Northwell Health, Zucker School of Medicine at Hofstra/Northwell, 270-05 76^th^ Avenue, Queens, NY 11040, United States

**Keywords:** Stress, Anxiety, COVID-19, Surgical delay, Peri-operative distress, Quality of life

## Abstract

**Background:**

Amid the height of the COVID-19 pandemic in the US, the US Surgeon General ordered hospitals and healthcare systems to stop all elective surgical procedures. The aim of our study was to evaluate the additional mental health impact of surgical delay on patients awaiting surgery for benign, pre-malignant and malignant conditions within the context of the COVID-19 pandemic.

**Study design:**

All patients over the age of 18 awaiting surgery for benign, pre-malignant or malignant conditions within the gynecologic oncology, surgical oncology and colorectal services across Northwell Health were eligible for participation. Upon successful enrollment, participants completed a baseline questionnaire consisting of the Generalized Anxiety Disorder Questionnaire, the Penn State Worry Questionnaire, and Brief-Illness Patient Questionnaire.

**Results:**

The surgical delay was considered moderately to extremely concerning by 72 % of survey respondents, with one third indicating the highest (10/10) level of concern. Fifty-five percent of patients with a pre-operatively suspected/confirmed cancer or pre-malignant condition demonstrated mild to severe anxiety in their completion of the GAD-7 scale. The average time awaiting surgery was 117 days (range 8–292); and 63 % of respondents indicated that the delay had a moderate to severe impact on their daily life.

**Conclusions:**

Patients awaiting surgery for confirmed, suspected or pre-malignant conditions expressed decreased sense of control and increased levels of distress compared to patients awaiting procedures for benign conditions (p < 0.05, 95 % CI [-2.65, -0.08]). Future research will focus on the effects of COVID-19 related delays in operative care on clinical outcomes, including cancer morbidity and mortality.

## Highlights

• Half of all cancer patients awaiting surgery during the COVID-19 pandemic screened positive for anxiety.
• Seventy-two percent of respondents indicated the delay was moderately to extremely distressing.
• The average time spent awaiting operative procedures during the COVID-19 pandemic was 117 days.

## 1 Introduction

At the beginning of March 2020, hospital admissions for COVID-19 positive patients began to increase at an unprecedented rate. As our New York based hospital system began to grapple with the prospect of surpassing capacity for inpatient admissions and patients requiring ICU and ventilator support, the possibility of canceling all elective surgery became an integral part of crisis discussions. This paralleled the conversation on the national stage. On March 14, 2020 the US Surgeon General asked hospitals and healthcare systems to consider stopping elective surgical procedures amid the COVID-19 pandemic in the US. On March 16th, all “elective” surgical procedures in New York City were halted under the emergency executive order; this decision was extended across the entirety of New York State on March 20th, 2020.

**Fig. 1:**
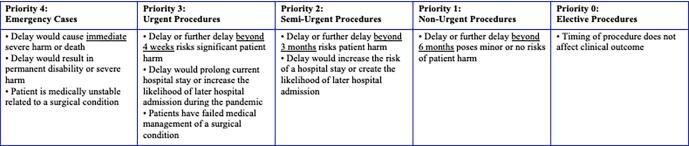
**Northwell Health Surgery Priority Scoring System. Caption:**Fig. 1 illustrates the criteria set forth by Northwell Health during the COVID-19 pandemic to prioritize and schedule surgical cases.

In an effort to assist in the triage of non-emergent surgical procedures, the American College of Surgeons (ACS) published the tiered Elective Surgery Acuity Scale (ESAS) (Covid-19, 2020). Within this three-tiered scale, Tier 1 represented low acuity surgical procedures performed on an outpatient basis in healthy or unhealthy patients. Tier 2 represented intermediate acuity surgeries performed for conditions not considered to be life threatening, but which have potential for future morbidity and mortality, and would require a hospitalization. Tier 1 and 2 surgical procedures were recommended to be postponed, or performed in an ambulatory surgery center. Tier 3 procedures represented the highest level of acuity, for patients requiring emergent surgery, not to be postponed. This encompassed most cancer surgeries, patients with severe symptoms, or other significant threats to life or limb. (Covid-19, 2020) However, given the extreme scarcity of available resources at the height of the pandemic in New York State, the majority of New York City and Long Island hospitals were unable to proceed with surgical procedures for pre-malignant and malignant conditions. At our institution, a priority scoring system was developed to triage beyond the American College of Surgeon’s classification of Tier 3 acuity (Fig. 1). Therefore, the majority of Tier 3 cases were postponed in addition to all non-emergent surgery.

Prior to the COVID-19 pandemic, it was reported that 47 % of outpatients with newly diagnosed cancer demonstrated symptoms satisfying DSM-III criteria for adjustment disorder with depressed mood, 25 % met criteria for mixed anxiety and depressed mood, 6 % for major depression, and 4 % for anxiety disorders. (Caruso et al., 2017) Amidst the initial stages of the COVID-19 pandemic, preliminary research demonstrated that 28.8 % of the general population in China reported moderate to severe anxiety symptoms due to the pandemic. (Wang et al., 2020) Our study aimed to understand the additional mental health impact of delayed operative care on patients awaiting surgery for benign, pre-malignant and malignant conditions in the context of the COVID-19 pandemic. We hypothesized that delay in operative care in our patient population caused a clinically significant increase in anxiety and worry, beyond what is typically experienced in the *peri*-operative setting.

## 2 Methods

The study was conducted across multiple hospitals at Northwell Health, the largest academic health system in New York. The Northwell Health institutional review board approved this study (IRB#: 20-0233) and recruitment began in May 2020. Patients were identified through a review of the surgical schedules of the departments of gynecologic oncology, colorectal surgery and surgical oncology across the health system. Payment was not provided for participation. Delay in operative care was defined as patients who had cancellation of their previously scheduled surgery as of March 14, 2020, and patients placed on a waiting list of surgeries needing to be scheduled once the operating rooms re-opened. Surgical cancellations and delays were captured between March 14, 2020 and June 30, 2020. Surgical delay was calculated from the time of cancelled surgery date to the date of surgery. Our eligible subject population included all patients over the age of 18, regardless of comorbidities, who were awaiting surgery for benign, pre-malignant or malignant conditions. Patients requiring rescheduling and new bookings were aware of indications for their surgeries and need for delay due to COVID Pandemic. Patients were classified as priority 3 given urgency of their procedures. Patients awaiting surgical management were notified of the study by phone and if interested, electronically signed an IRB approved consent via REDCap. Upon their successful enrollment in the study, patients were asked to complete a baseline questionnaire consisting of demographics questions and the following valid scales: Generalized Anxiety Disorder Questionnaire (GAD-7), the Penn State Worry Questionnaire, and Brief-Illness Patient Questionnaire. These questionnaires are based on a Likert scale and scores were calculated based on each individual validated scoring system. Study data were collected electronically via e-mail and managed using REDCap electronic data tools hosted at Northwell. (Harris et al., 2009, Harris et al., 2019).

The GAD-7 is a research-validated and frequently utilized screening questionnaire for anxiety. It is 89 % sensitive in the diagnosis of generalized anxiety disorder, with a specificity of 82 %. (Löwe et al., 2008) The Penn State Worry Questionnaire is an independent, 16 item instrument which possesses a high consistency and test–retest reliability with respect to delineating worry. (Meyer et al., 1990) The Brief-Illness Patient Questionnaire is a 9-item instrument demonstrated to assess the cognitive and emotional impact of illness; providing a rapid assessment of patients’ illness perception. The scale has been demonstrated to have good predictive validity with respect to mental and physical function at 3-month follow-up dates. (Broadbent et al., 2006) Statistical analysis was performed via student’s *t*-test and two-way ANOVA with a p-value of<0.05 deemed significant.

## 3 Results

Of the 915 patients awaiting surgical care within the gynecologic oncology, surgical oncology and colorectal surgery services at Northwell Health, 637 patients were contacted and 163 patients consented to participate in the study. Of the 163 patients who completed the surveys: 31 % were gynecologic oncology patients, 48 % surgical oncology, and 21 % of patients were awaiting procedures within the colorectal surgery division. Of the 163 participants; pre-operatively, 31 % (50/163) had a pathologically confirmed malignancy and 45 % (73/163) had a suspected benign condition. An additional 8 % (14/163) were awaiting surgery for a pre-malignant condition and 16 % (26/163) were awaiting surgery for a condition which was suspected to be cancer (Table 1). At the time of this manuscript submission, 123 of 163 patients had undergone their intended surgical procedure. The average time spent waiting for surgery was 117 days (range: 8–292). Postoperatively, 38 % (47/123) of patients had a pathologically proven cancer, 12 % (14/123) were diagnosed with a pre-malignant condition, and pathology for the remaining 50 % (62/123) demonstrated benign conditions.

**Table 1:** Patient Characteristics.

			n = 163	%
**Race**				
	White		107	65
	Black		28	17
	American Indian/ Alaskan Native		1	0
	Asian		13	8
	Native Hawaiian/ Other Pacific Islander		0	0
	Other/Prefer not to answer		17	10
**Gender** Female Male			135 28	83 17
**Hispanic or Latino**				
	Yes		22	14
	No		137	84
	Prefer not to answer		4	2
**Pre-Operative Diagnosis**				
	Suspected benign		73	45
	Pre-malignant		14	8
	Suspected cancer		26	16
	Cancer		50	31
**Surgical Fsubspecialty**				
	Gynecologic Oncology		50	31
	Surgical Oncology		79	48
	Colorectal		34	21
**Marital Status**				
	Now married		84	51
	Widowed		9	6
	Divorced		21	13
	Separated		3	2
	Never married		46	28
**Chronic Health Conditions**				
	Diabetes		15	9
	Hypertension		47	29
	Heart Disease		6	4
	Respiratory Disease (Asthma, COPD, Chronic Bronchitis)		19	12
	Other		27	17
	History of Depression or Anxiety		41	25
**Highest Level of Education**				
	Some high school, but no degree		7	4
	High school diploma		38	23
	Vocational training after high school, other than college		11	7
	College degree		72	44
	Post-graduate degree		34	21
**Current Employment Status**				
	Employed full-time		83	51
	Employed part-time		16	10
	Not employed		29	18
		Unemployed prior to COVID-19 pandemic	16	
		Unemployed 2/2 COVID-19 pandemic	12	
	Retired		35	21

***Caption:***Table 1*provides a breakdown of demographic and diagnosis specific attributes of our patient population.*

**Fig. 2:**
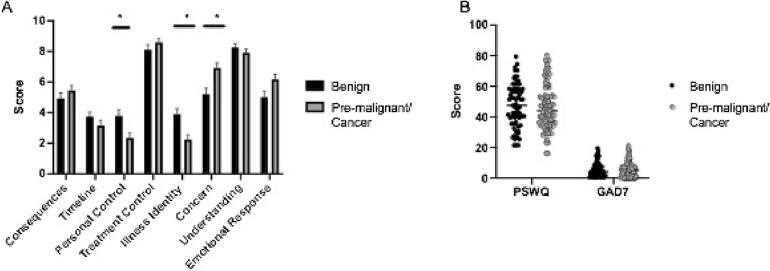
**Patient survey results stratified by pre-operative diagnosis. Caption:**Fig. 2 demonstrates variation in the results of the B-IPQ (A), PSWQ and GAD-7 (B) amongst patients with benign versus malignant pre-operative diagnoses. A p value of < 0.05 was considered statistically significant.

**Fig. 3:**
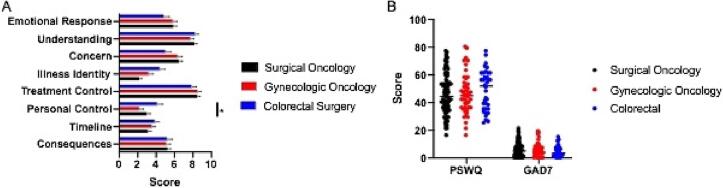
**Patient survey results stratified by surgical service line. Caption:**Fig. 3 demonstrates variation in the results of the B-IPQ (A), PSWQ and GAD-7 (B) amongst patients receiving treatment within the gynecologic oncology, surgical oncology and colorectal surgical service lines. A p value of < 0.05 was considered statistically significant.

Utilizing the B-IPQ questionnaire, surgical delay was considered moderately to extremely concerning by 72 % (117/163) of respondents; with one-third of respondents endorsing the highest level of concern (10/10) on a 10-point scale, indicating a very high level of distress. Patients with confirmed, suspected or pre-malignant conditions expressed a statistically significant increased level of concern surrounding the surgical delay compared to patients awaiting procedures for benign conditions (p < 0.05, 95 % CI [−2.65, −0.08]) (Fig. 2A, Supplemental Table 1). Sixty-three percent (n = 103/163) of participants indicated that the surgical delay had a moderate to severe effect on their daily life, with 25 % (n = 41/163) indicating a severe effect on their daily life. The majority of respondents (66 %, n = 108/163) indicated the sensation of having little to no control over their illness; and nearly one-third of patients (30 %, n = 50/163) indicated experiencing moderate to severe illness symptoms. Additionally, patients awaiting procedures for benign conditions expressed an increased level of personal control over their situation than those patients awaiting surgical procedures for confirmed, suspected, or pre-malignant conditions (Fig. 2A, Supplemental Table 1). A total of 82 % (n = 135/163) of patients felt they understood their illness well. Patients awaiting operations for benign conditions endorsed a lesser symptom burden (illness identity) than those with suspected/confirmed or pre-malignant conditions (Fig. 2A, Supplemental Table 1). Ultimately, 81 % (n = 132/163) of all the patients were optimistic that the delay would be only short-term. When asked if patients would be willing to undergo procedures during the pandemic, 82 % (n = 133/163) of patients responded that if given the opportunity to reschedule their surgical procedure within the next month, they would have the procedure despite the ongoing pandemic. When evaluating the data by surgical subspecialty, gynecologic oncology patients exhibited less personal control of their illness vs. colorectal surgery patients (p < 0.05, 95 % CI [-3.49, −0.37]) (Fig. 3A, Supplemental Table 2).

The GAD-7 responses across the entire study population indicated that 15 % (n = 22/148) met criteria for moderate to severe anxiety with an additional 28 % (n = 42/148) meeting criteria for mild anxiety. The remaining 57 % (n = 84/148) did not demonstrate significant anxiety in their responses. However, 50 % (n = 40/80) of all patients with a preoperatively suspected or confirmed cancer, or a pre-malignant condition, demonstrated mild to severe anxiety in their GAD-7 response. Within this subset of patients, 38 % (n = 15/40) met criteria for moderate-to-severe anxiety, while the remaining 62 % (n = 25/40) demonstrated mild anxiety. There was no significant difference in levels of pre-operative anxiety among the patients with benign disease when compared to patients with pre-malignant disease or cancer (Fig. 2B, Supplemental Table 1). In evaluating anxiety by surgical service, 50 % (n = 16/32) of gynecologic oncology patients, 48 % (n = 37/77) of surgical oncology patients, and 38 % (n = 12/32) of colorectal patients experienced some level of anxiety. These slight variations among surgical subspecialty were not statistically significant (Fig. 3B, Supplemental Table 2). Within the subset of gynecologic oncology patients: 75 % (n = 12/16) experienced mild anxiety, and 25 % (n = 4/16) experienced symptoms consistent with either moderate or severe anxiety.

Among the participants’ response to the Penn State Worry Questionnaire: 19 % (n = 29/152) demonstrated high worry, 45 % (n = 68/152) demonstrated moderate worry, and the remaining 36 % (n = 55/152) demonstrated low worry. With respect to the experience of worry, preoperative diagnoses of benign, confirmed or suspected malignant, and pre-malignant conditions were fairly evenly distributed and there was no statistically significant difference (Fig. 2B, Supplemental Table 1). There was also no statistically significant difference in experience of worry among patients of the three different surgical services (Fig. 3B, Supplemental Table 2). Across the study cohort, there were no differences noted in any B-IPQ subgroups, the GAD-7 score, and the PSWQ score based on race, marital status, or employment status (p > 0.05).

## 4 Discussion

To our knowledge, this is the first study in the literature to focus on patient anxiety and worry due to delay in surgical care during the COVID-19 pandemic. Existing research has focused largely on the logistics and safety of performing surgical procedures during the pandemic, with little attention to the impact of broadly implemented surgical holds on patient psychological distress. (Kimmig et al., 2020, Bartlett et al., 2020 Jun, Finley et al., 2020 May 1) Additional research has focused upon patient perception of surgical delay, and willingness to reschedule surgery. (Brown et al., 2020, Sii et al., 2020 Oct) A study investigating patients’ attitudes towards the postponement of scheduled procedures for pelvic floor disorders discovered that up to half of patients were upset by the delay in their care (Mou et al., 2020), but did not quantify the impact on patient psyche. Research has determined when faced with delay in ovarian cancer care due to the COVID-19 pandemic, 51.4 % of patients experienced borderline or abnormal anxiety, and 88.6 % experienced significant worry. (Frey et al., 2020) However, the delay in care evaluated by the aforementioned study ranged from delayed imaging studies and laboratory results, to physician visits in a very specific patient population. This study focuses specifically on delay in operative procedures, and evaluates a more diverse patient population encompassing different surgical services.

Patients experiencing delays in their planned surgical procedures demonstrated significant distress. Although it is suspected that the COVID-19 pandemic affects levels of anxiety and worry throughout the general population; one-third of study respondents indicated a level of 10 on a 10-point scale of concern with specific respect to the delay in their surgical care. Most notably, 72 % of respondents indicated the delay was moderately to extremely disturbing. Existing research evaluating the psychological impact of COVID-19 on the general population in China had previously identified 53.8 % of the population as reporting the psychological impact of the outbreak as moderate to severe. (Wang et al., 2020) Our findings indicate an increased level of anxiety and stress experienced by *peri*-operative patients experiencing quantifiable and notable delays in surgical care. The study population was composed of patients awaiting surgical procedures with colorectal, surgical oncology or gynecologic oncology surgeons, and included a near equal mix of suspected benign and malignant conditions. Patients awaiting surgical procedures for suspected, confirmed, or pre-malignant conditions exhibited a statistically significant increased level of concern surrounding the delay compared to those awaiting surgical procedures for benign conditions. Interestingly, concurrent research identified that couples undergoing assisted reproduction treatment, postponed at the time of the COVID-19 outbreak, experienced increased psychological distress and anxiety. (Esposito et al., 2020, Tokgoz et al., 2020 Aug 19, Barra et al., 2020) This was also seen in both the elderly population and those who had surgical delays outside of the United States. (Loh et al., 2022, Sauro et al., 2023). This highlights the generalizable distress felt by those experiencing delays during the pandemic.

Although sample size was not calculated prior to initiating data collection, post-hoc calculation demonstrates that this study is powered adequately to detect increased anxiety and worry among our study population, as evidenced by their GAD-7 and PSWQ score. A limitation of this study is the short follow up interval, which makes it difficult to clearly identify the impact of the surgical delay on long-term clinical patient outcomes. Another limitation of this study is a low response rate of 26 %, which is likely explained by several factors. We did not exclude a large cohort of patients, who we were unable to contact via telephone after three attempts, but rather sent the survey to their email address on file. Furthermore, our response rate was impacted by the logistics of conducting an email-based survey in an urban based, elderly population. Prior research has determined decreased response to email surveys, and an average response rate to online surveys of 46 % across all populations. (Sauro et al., 2023, Zha et al., 2020 Mar) This may be exacerbated by the additional findings that elderly and urban populations are likely to have decreased survey response rates. (Meyer et al., 2020) The major strength of this study is that it is the largest reported cohort of patients with delayed operative care during the COVID-19 pandemic. This study delineates the psychological impact of delay in care on *peri*-operative patients at the time of their delay. However, it is difficult to discern if the extent of reported levels of anxiety, worry and distress are specifically related to delayed operative care, versus the anxiety and worry induced by the COVID-19 pandemic. Patients with a history of anxiety, depression or other psychiatric illness were not excluded from the study. Regardless, the prevalence of anxiety in this study, specifically anxiety experienced by patients with cancer, surpasses previously reported data in the literature for both *peri*-operative patients (Coste et al., 2013) and preliminary reports of COVID-19 related anxiety amongst all patients in China. (Wang et al., 2020) Further research, including longer term follow-up, will be necessary to comprehensively understand the impact of the surgical delays on the clinical outcomes, including cancer morbidity and mortality.

It is important to consider the disproportionate effect that pandemic-related delays in care may have on pre-operative patients, especially those patients with cancer. Despite the majority of cancer surgery being classified by the ACS as not to be postponed, in times of extreme stress upon healthcare resources, even cancer surgery may intermittently be categorized as elective, as was evident during the first COVID-19 outbreak in New York State. (Fig. 1) The catastrophic disaster of the pandemic in New York State was highlighted by the differences in treatment of cancer patients as compared to other states in the U.S. As health care systems plan for future COVID-19 resurgences or other national disasters, it is necessary to consider the emotional impact of operative delay and create strategies to guide and support our surgical patients. It is prudent that clinical care teams offer specific support to patients awaiting surgical management. There is great clinical significance to the increased distress, decreased sense of control and increased symptom burden experienced by the patients with cancer. Further research is warranted to evaluate the clinical impact of early identification and treatment of perioperative anxiety and long-term outcomes of operative delays on cancer care. It is imperative for clinicians to consider that while the psychological impact of operative delay is a fact, it may vary significantly by pre-operative suspicion or confirmation of malignancy. Patients at risk for high *peri*-operative distress may benefit from additional support and counseling throughout the *peri*-operative period. Future research will focus on clinical outcomes of patients with surgical delay as well as long-term psychologic effects of delay of treatment in cancer patients. Our focus should address the specific needs of delay of surgery in cancer patients – such as setting up additional resources for this vulnerable population.

### CRediT authorship contribution statement

**Michelle A. Soloff:** Data curation, Formal analysis, Writing – original draft, Writing – review & editing. **Trey Keel:** Data curation. **Aaron Nizam:** Formal analysis, Writing – review & editing. **Gary L. Goldberg:** Conceptualization, Methodology, Resources, Writing – review & editing. **Antoinette Sakaris:** Writing – review & editing. **Michael A. Diefenbach:** Conceptualization, Writing – review & editing. **Danielle K. DePeralta:** Conceptualization, Writing – review & editing. **Marina Frimer:** Conceptualization, Formal analysis, Investigation, Methodology, Resources, Supervision, Validation, Writing – review & editing.

## Declaration of Competing Interest

The authors declare that they have no known competing financial interests or personal relationships that could have appeared to influence the work reported in this paper. No funding/grant provided for this research.
