# Supplementary material for: Stress, anxiety, and illness perception in patients experiencing delay in operative care due to the COVID-19 pandemic
Source: Gynecol Oncol Rep. 2023 Jul 13;48:101245. doi: 10.1016/j.gore.2023.101245 (PMC10422101; doi:10.1016/j.gore.2023.101245)
Supplement: Supplementary data 1 [file mmc1.docx]

**Supplemental Table 1:** 2-way ANOVA of patient survey results, stratified by pre-operative diagnosis

| **B-IPQ** | | | | | |
| --- | --- | --- | --- | --- | --- |
|  | **Suspect benign** | **Suspect malignant** | **DIFFERENCE** | **95.00% CI** | **Adjusted P Value** |
| Consequences | 5.069 | 5.368 | -0.2995 | -1.583 to 0.9843 | 0.9974 |
| Timeline | 3.713 | 3.08 | 0.6326 | -0.6557 to 1.921 | 0.7957 |
| Personal Control | 3.437 | 2.487 | 0.9499 | -0.3339 to 2.234 | 0.2992 |
| Treatment Control | 8.058 | 8.711 | -0.6524 | -1.940 to 0.6349 | 0.7671 |
| Illness Identity | 3.552 | 2.316 | 1.236 | -0.04787 to 2.520 | 0.0671 |
| ****Concern** | **5.517** | **6.882** | **-1.364** | **-2.648 to -0.08054** | **0.0297** |
| Understanding | 7.931 | 8.197 | -0.2663 | -1.550 to 1.017 | 0.9989 |
| Emotional Response | 5.276 | 6.079 | -0.8031 | -2.087 to 0.4807 | 0.5204 |
|  | | | | | |
| **PSWQ** | | | | | |
|  | **Suspect Benign** | **Suspect Malignant** | **DIFFERENCE** | **95.00% CI** | **Adjusted P Value** |
|  | 47.3 | 45.77 | 1.524 | -2.373 to 5.422 | 0.6158 |
|  | | | | | |
| **GAD-7** | | | | | |
|  | **Suspect Benign** | **Suspect Malignant** | **DIFFERENCE** | **95.00% CI** | **Adjusted P Value** |
|  | 5.296 | 5.757 | -0.4605 | -4.406 to 3.485 | 0.9573 |
|  |  |  |  |  |  |
| **Statistically significant result | | | | | |
|  |  |  |  |  |  |
|  |  |  |  |  |  |

***Caption: Supplemental*** *Table 1 demonstrates a comparison of survey results between patients with suspected benign versus malignant pre-operative diagnoses.*

**Supplemental Table 2:** 2-way ANOVA of patient survey results, stratified by surgical service line

| **B-IPQ** |  |  |  |  |  |
| --- | --- | --- | --- | --- | --- |
|  | **Predicted mean (A)** | **Predicted mean (B)** | **Predicted mean diff.** | **95.00% CI** | **Adjusted P Value** |
| **Consequences** |  |  |  |  |  |
| Surgical Oncology (A) vs. Gynecologic Oncology (B) | 5.241 | 5.14 | 0.1005 | -1.169 to 1.370 | 0.9811 |
| Surgical Oncology (A) vs. Colorectal (B) | 5.241 | 5.235 | 0.005212 | -1.435 to 1.446 | >0.9999 |
| Gynecologic Oncology (A) vs. Colorectal (B) | 5.14 | 5.235 | -0.09529 | -1.656 to 1.466 | 0.9888 |
|  |  |  |  |  |  |
| **Timeline** |  |  |  |  |  |
| Surgical Oncology (A) vs. Gynecologic Oncology (B) | 3.128 | 3.54 | -0.4118 | -1.684 to 0.8605 | 0.728 |
| Surgical Oncology (A) vs. Colorectal (B) | 3.128 | 3.912 | -0.7836 | -2.227 to 0.6597 | 0.4102 |
| Gynecologic Oncology (A) vs. Colorectal (B) | 3.54 | 3.912 | -0.3718 | -1.933 to 1.189 | 0.842 |
|  |  |  |  |  |  |
| **Personal Control** |  |  |  |  |  |
| Surgical Oncology (A) vs. Gynecologic Oncology (B) | 2.987 | 2.22 | 0.7673 | -0.5018 to 2.037 | 0.3315 |
| Surgical Oncology (A) vs. Colorectal (B) | 2.987 | 4.147 | -1.16 | -2.600 to 0.2808 | 0.1422 |
| ****Gynecologic Oncology (A) vs. Colorectal (B)** | **2.22** | **4.147** | **-1.927** | **-3.488 to -0.3659** | **0.0107** |
|  |  |  |  |  |  |
| **Treatment Control** |  |  |  |  |  |
| Surgical Oncology (A) vs. Gynecologic Oncology (B) | 8.474 | 8.52 | -0.04564 | -1.318 to 1.227 | 0.9961 |
| Surgical Oncology (A) vs. Colorectal (B) | 8.474 | 7.882 | 0.592 | -0.8513 to 2.035 | 0.6008 |
| Gynecologic Oncology (A) vs. Colorectal (B) | 8.52 | 7.882 | 0.6376 | -0.9235 to 2.199 | 0.6034 |
|  |  |  |  |  |  |
| **Illness Identity** |  |  |  |  |  |
| Surgical Oncology (A) vs. Gynecologic Oncology (B) | 2.177 | 3.24 | -1.063 | -2.332 to 0.2064 | 0.1214 |
| ****Surgical Oncology (A) vs. Colorectal (B)** | **2.177** | **4.441** | **-2.264** | **-3.704 to -0.8235** | **0.0007** |
| Gynecologic Oncology (A) vs. Colorectal (B) | 3.24 | 4.441 | -1.201 | -2.762 to 0.3600 | 0.1682 |
|  |  |  |  |  |  |
| **Concern** |  |  |  |  |  |
| Surgical Oncology (A) vs. Gynecologic Oncology (B) | 6.481 | 6.38 | 0.101 | -1.168 to 1.370 | 0.981 |
| Surgical Oncology (A) vs. Colorectal (B) | 6.481 | 5.059 | 1.422 | -0.01831 to 2.863 | 0.0539 |
| Gynecologic Oncology (A) vs. Colorectal (B) | 6.38 | 5.059 | 1.321 | -0.2400 to 2.882 | 0.1161 |
|  |  |  |  |  |  |
| **Understanding** |  |  |  |  |  |
| Surgical Oncology (A) vs. Gynecologic Oncology (B) | 8.165 | 7.74 | 0.4246 | -0.8446 to 1.694 | 0.7124 |
| Surgical Oncology (A) vs. Colorectal (B) | 8.165 | 8.265 | -0.1001 | -1.541 to 1.340 | 0.9854 |
| Gynecologic Oncology (A) vs. Colorectal (B) | 7.74 | 8.265 | -0.5247 | -2.086 to 1.036 | 0.7101 |
|  |  |  |  |  |  |
| **Emotional Response** |  |  |  |  |  |
| Surgical Oncology (A) vs. Gynecologic Oncology (B) | 5.873 | 5.84 | 0.03342 | -1.236 to 1.303 | 0.9979 |
| Surgical Oncology (A) vs. Colorectal (B) | 5.873 | 4.853 | 1.02 | -0.4200 to 2.461 | 0.2202 |
| Gynecologic Oncology (A) vs. Colorectal (B) | 5.84 | 4.853 | 0.9871 | -0.5741 to 2.548 | 0.2991 |
|  |  |  |  |  |  |
| **PSWQ** |  |  |  |  |  |
|  | **Predicted mean (A)** | **Predicted mean (B)** | **Predicted mean diff.** | **95.00% CI** | **Adjusted P Value** |
| Surgical Oncology (A) vs. Gynecologic Oncology (B) | 45.71 | 46.15 | -0.4407 | -5.141 to 4.259 | 0.9735 |
| Surgical Oncology (A) vs. Colorectal (B) | 45.71 | 48.92 | -3.214 | -8.328 to 1.901 | 0.302 |
| Gynecologic Oncology (A) vs. Colorectal (B) | 46.15 | 48.92 | -2.773 | -8.378 to 2.832 | 0.475 |
|  |  |  |  |  |  |
| **GAD-7** |  |  |  |  |  |
|  | **Predicted mean (A)** | **Predicted mean (B)** | **Predicted mean diff.** | **95.00% CI** | **Adjusted P Value** |
| Surgical Oncology (A) vs. Gynecologic Oncology (B) | 6.026 | 5.234 | 0.7923 | -3.962 to 5.547 | 0.9187 |
| Surgical Oncology (A) vs. Colorectal (B) | 6.026 | 4.667 | 1.36 | -3.824 to 6.543 | 0.8106 |
| Gynecologic Oncology (A) vs. Colorectal (B) | 5.234 | 4.667 | 0.5674 | -5.107 to 6.242 | 0.9699 |
|  |  |  |  |  |  |
| **Statistically significant result |  |  |  |  |  |
|  |  |  |  |  |  |
|  |  |  |  |  |  |

***Caption: Supplemental*** *Table 2 demonstrates a comparison of survey results between surgical service lines*
